# Discovery and validation of novel biomarkers for detection of cervical cancer

**DOI:** 10.1002/cam4.3799

**Published:** 2021-02-23

**Authors:** Zigang Li, Jianhua Chen, Shaobo Zhao, Yajun Li, Jie Zhou, Jianghong Liang, Huifang Tang

**Affiliations:** ^1^ Department of Anesthesiology Women’s Hospital School of Medicine Zhejiang University Hangzhou China; ^2^ Department of Pathology Women’s Hospital School of Medicine Zhejiang University Hangzhou China; ^3^ Department of Pharmacology School of Basic Medical Sciences Zhejiang University Hangzhou Zhejiang China; ^4^ Department of Anesthesiology Tongde Hospital of Zhejang Province Hangzhou China; ^5^ Department of Stomatology Guangzhou Hospital of Integrated Traditional and West Medicine Guangzhou China

**Keywords:** biomarker, cervical cancer, CRISP2, GEO, KRT17

## Abstract

**Aims:**

To investigate novel biomarker for diagnosis of cervical cancer, we analyzed the datasets in Gene Expression Omnibus (GEO) and confirmed the candidate biomarker in patient sample.

**Materials and methods:**

We collected major datasets of cervical cancer in GEO, and analyzed the differential expression of normal and cancer samples online with GEO2R and tested the differences, then focus on the GSE63514 to screen the target genes in different histological grades by using the R‐Bioconductor package and R‐heatmap. Then human specimens from the cervix in different histological grades were used to confirm the top 8 genes expression by immunohistochemical staining using Ki67 as a standard control.

**Results:**

We identified genes differentially expressed in normal and cervical cancer, 274 upregulated genes and 206 downregulated genes. After intersection with GSE63514, we found the obvious tendency in different histological grades. Then we screened the top 24 genes, and confirmed the top 8 genes in human cervix tissues. Immunohistochemical (IHC) results confirmed that keratin 17 (KRT17) was not expressed in normal cervical tissues and was over‐expressed in cervical cancer. Cysteine‐rich secretory protein‐2 (CRISP2) was less expressed in high‐grade squamous intraepithelial lesions (HSILs) than in other histological grades.

**Conclusion:**

For the good repeatability and consistency of KRT17 and CRISP2, they may be good candidate biomarkers. Combined analysis of KRT17, CRISP2 expression at both genetic and protein levels can determine different histological grades of cervical squamous cell carcinoma. Such combined analysis is capable of improving diagnostic accuracy of cervical cancer.

## Graphical abstract

To investigate novel biomarker for diagnosis of cervical cancer, we analysed the datasets in Gene Expression Omnibus (GEO) and confirmed the candidate biomarker in patient sample. And we found the good repeatability and consistency of KRT17 and CRISP2, they may be good candidate biomarkers. Combined analysis of KRT17, CRISP2 expression at both genetic and protein levels can determine different histological grades of cervical squamous cell carcinoma. Such combined analysis is capable of improving diagnostic accuracy of cervical cancer.

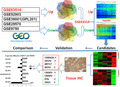

## 1 INTRODUCTION

Cervical cancer is one of the leading causes of death in women worldwide, with 12,990 new cases and 4120 cancer deaths reported in the United States in 2016.^1^ The development of cervical cancer is a multi‐step biological process initiated by a series of genetic and transcriptional changes. Cervical cancer in early stages is overwhelmingly likely to be cured through a combined treatment of surgery, radiotherapy or chemotherapy. In contrast, options for patients in late stages involve alternative treatment such chemo‐radiotherapy, with a poor prognosis.^2^ Therefore, diagnosing cervical cancer at an early stage allows for effective treatment and substantially improves outcomes. In nearly all cases, human papilloma virus (HPV) infection is the primary risk factor for the development of cervical cancer, and HPV16 and HPV18 account for 70% of cervical cancer and precancerous cervical lesions.^3^ Although routine Pap smears and HPV tests have largely improved the outcome of cervical cancer in developed countries, in many developing areas, the disease is often not identified until advanced stages.^4^ Furthermore, HPV tests have low specificity for cervical cancer because many HPV infections are transient and are not prone to malignant transformation.^5^ Squamous cell carcinoma antigen (SCC‐Ag) is used clinically to predict squamous cell carcinoma (SCC); however, it lacks specificity for cervical cancer.^6^ Therefore, identification of specific biomarkers remains an urgent need for increasing diagnostic accuracy.

In this study, to distinguish specific biomarkers among different histological grades, we first collected all datasets in the Gene Expression Omnibus (GEO) database, and select the analyzed a microarray (NO: GSE63514), and then confirmed the results by immunohistochemical (IHC) staining in specimens from patients. This microarray included 24 normal specimens, 14 cervical intraepithelial neoplasia 1 (CIN1) lesions, 22 CIN2 lesions, 40 CIN3 lesions and 28 cancer specimens, and found top 24 candidate biomarkers which were highly or lowly expressed in different histological grades, with KRT17 and CRISP2 being the most significant. The further IHC results suggested that KRT17 was dominantly expressed in the cervical squamous cancer and barely expressed in the normal cervix; while CRISP2 was specifically lowly expressed in HSIL. Combined with Ki67 is gradual up‐regulation with tumor progression,^7^ our results indicated that KRT17 and CRISP2 are possible biomarkers for different histological stages of cervical squamous cancer, this findings that may provide new targets for mechanistic studies and adjuvant diagnosis or therapy for cervical cancer.

## 2 MATERIALS AND METHODS

### 2.1 Cervical cancer microarray acquisition from the GEO database

To find cervical cancer related gene chip data, we collected all sets in GEO database of the National Center of Biotechnology Information (NCBI), the search query “("uterine cervical neoplasms"[MeSH Terms] OR cervical cancer [All Fields]) AND (“gse”[Filter] AND "Expression profiling by array"[Filter] AND (“10” [n_samples]: “1000”[n_samples])” was used to select the series.

### 2.2 Data extraction and differential gene expression analysis between normal and cervical cancer

For comparisons among normal and cancer tissues, we analyzed all genes of normal and cancer samples online with GEO2R and tested the differences between the two categories in every dataset we selected, after then we downloaded corresponding files to screen differentially expressed genes, respectively. Then drawing two venn diagrams of genes with significantly (adjustment *p* value<0.05) high and low expression in five datasets with the largest total sample size, and listing the expression in the five datasets above of these genes as the result, which was draw with an online tool on a website specialized to make venn diagrams (http://bioinformatics.psb.ugent.be/webtools/Venn/).

### 2.3 Differential gene expression analysis of various histological grade of cervical cancer

In the all GEO datasets, only one dataset (registration number GSE63514, originated from the University of Wisconsin–Madison, the National Cancer Institute and the University of Oklahoma Health Sciences Center) includes different histological grades of cervical: 24 normal specimens, 14 CIN1 lesions, 22 CIN2 lesions, 40 CIN3 lesions and 28 cancer specimens.^8^ The female patients had been recruited into Study to Understand Cervical Cancer Early Endpoints and Determinants (SUCCEED); the average age was 31.7, 67% were white, 15% were African‐American, 5% were Indian/Alaskan Natives, 1% were Asian and 15% were Hispanic. Specimens that were initially classified as CIN1 were reclassified as LSIL and specimens that were classified as CIN2 or CIN3 were reclassified as HSIL. All other specimens were classified as originally reported without revision of the initial diagnoses.

In order to further explore the regularity of some possible key genes during the progression from normal cervix to cervical cancer, we extracted the dataset of GSE63514 on GEO Datasets, then respectively gathered intersections of five datasets above in venn diagrams and top 100 genes (here we only analysed individual probes) with significantly (adjustment *p* value <0.05) high or low expression in GSE63514 as possible key genes, and used MEV 4.9.0 (www.tm4.org) to produce heatmaps to analyse the expression of these possible key genes among five categories that represent different periods of cervical cancer: normal, CIN I, CIN II, CIN III, SCC. Meantime hierarchical clustering analysis of genes based on Euclidean distance was conducted with MEV 4.9.0.

Using R statistical programming and BiocLite of Bioconductor ^9^ to assess differences in gene expression, we summarized probe‐level data to log2‐scale values and identified the strongest changes associated with each histopathological tissue class. The log2 values were decomposed as a sum of fixed and random effects. The result of differential expression analysis was the logarithm of the gene expression changes (logFC) in the experimental group multiplied by the control group. The *t*‐test was performed for statistical analysis of variance. The absolute logFC value of the difference between the two groups >1 and *p* < 0.05 was the standard for significantly differential expression, and the top 24 up‐ or down‐regulated genes during carcinogenesis were revealed via R‐heatmap for cluster analysis. At last, take advantage of Panther (http://www.pantherdb.org) to functional cluster analysis between mutual groups from controls, CIN1,2,3 to cancers.

### 2.4 Resource of cervical tissue specimens

To validate the results of genetic analysis at the transcriptional level, human cervical tissues were collected from the outpatient or surgical divisions of the Department of Gynaecology in Women's Hospital School of Medicine Zhejiang University between June and December 2017. We collected a total of 69 specimens for analysis according to the 4th Edition of WHO Classification of Tumours of the Female Reproductive Organs (IARC WHO Classification of Tumours). The histopathologic diagnosis was made by five pathologists and included normal tissues in 15 patients, LSIL in 19 patients, HSIL in 18 patients and SCC in 17 patients. We excluded patients with any other malignancies or a history of preoperative anticancer treatment. Unstained paraffin sections from all specimens were prepared to do IHC staining, after which the specimens were diagnosed again by the same pathologists.

### 2.5 Immunohistochemical (IHC) staining

The IHC staining was performed as follows. First, paraffin sections were treated twice with xylene for 10 min each, re‐hydrated through 100%, 95%, 85% and 75% ethanol each for 5 min and washed three times with PBS. The slices were incubated in 3% hydrogen peroxide for 10 min to remove endogenous peroxidase, washed three times with PBS, boiled in 10 mM sodium citrate buffer solution (pH 6.0) for 20 min for antigen retrieval and washed three times with PBS after bringing to room temperature. After blocking the nonspecific binding site with 5% bovine serum albumin (BSA) for 10 min, the slices were incubated with a specific antibody (as shown in Table 1) overnight at 4°C. The slices were then washed three times with PBS and incubated for 30 min at room temperature with a sheep anti‐mouse or anti‐rabbit antibody conjugated with HRP (Boster, Wuhan, China) (1:100 dilution in 1% BSA in PBS). The slices were then washed three times with PBS and treated with 3’3‐diaminobenzidine tetra‐hydrochloride (DAB) followed by counterstaining with haematoxylin for 5 min. After being dehydrated through 75%, 85%, 95% and 100% ethanol for 5 min each and incubated in xylene for 10 min twice, the slices were finally mounted with neutral gum. The tissue sections were observed under an Olympus microscope (Olympus Corporation, Japan) and images were taken at 200× magnification using the same light intensity and exposure time. Before we examined the images, we excluded non‐lesional contamination and nonspecific staining sections based on the judgments of the five certified pathologists. Image‐Pro Plus (IPP) image analysis software (Media Cybernetics, USA) was used to analyse the integrated optical density (IOD) value of each image, blinded to the corresponding clinical data.

**TABLE 1:** Primary antibody used in the study

Gene name	Antibody (company‐catlog)	Dilution
Ki67	Gentex‐ GX16667	1:100
CDKN2A	Santa cruz ‐Sc−73434	1:50
SYCP2	Santa cruz ‐Sc−20841	1:50
KRT17	Santa cruz ‐Sc−393091	1:25
NEFH	Santa cruz ‐Sc−22909	1:25
CRISP3	Santa cruz ‐Sc−377505	1:25
CRISP2	Abnova PAB19289/	1:100
	Santa cruz ‐Sc−390914	1:50
DSG1	Santa cruz ‐Sc−20114	1:25
PPP1R3C(PTG)	Santa cruz ‐Sc−6582	1:50

### 2.6 Statistical analysis

The absolute logFC value of the difference between the two group >1, and *p* < 0.05 is the standard of significantly differential expression. Due to the heterogeneity of IOD variance in different groups, the post hoc test was performed for the IHC results. Error bars for the experiments represent the standard deviation of the mean value (mean value ±S.D.). SPSS24 software was used to analyse all IHC results and *p* < 0.05 was regarded as statistically significant.

## 3 RESULTS

### 3.1 Cervical cancer in GEO datasets

To investigate the cervical cancer in GEO, we collected all results in GEO database listed in Table 1. The key words we used to search in GEO datasets: "uterine cervical neoplasms"[MeSH Terms] OR cervical cancer [All Fields]) AND ("gse"[Filter] AND "Expression profiling by array"[Filter]. There are total 17 datasets in GEO database. All datasets have two groups: normal and cancer, common used datasets were GPL6244 and GPL570, only one dataset, GSE63514 contains the all grades of cervical cancer from normal, CIN 1~III, to SCC. Meanwhile, in all datasets, the sample size is another obstacle, for the smaller size limited the further analysis, so, in the next step, we selected five datasets which the minimum sample size beyond 50, marked by red in Table 2.

**TABLE 2:** Cervical cancer in GEO datasets (updated by July 6, 2019)

Order	GSE number	Platforms	NORMAL	CANCER	CIN I	CIN II	CIN III
1	89657	GPL6244	3	4			
2	75132	GPL570	21	1			
3	63678	GPL571	5	5			
4	63514	GPL570	24	28	14	22	40
5	52903	GPL6244	17	55			
6	67522	GPL10558	22	20			
7	46857	GPL7025	4	25			
8	39001	GPL201	12	43			
9		GPL6244	5	19			
10	29570	GPL6244	17	45			
11	14404	GPL6699	5	28	7		
12	9750	GPL96	24	33			
13	7410	GPL1708	5	40			
14	7803	GPL96	10	21			
15	6791	GPL570	8	20			
16	4482	GPL3515	3	9			
17	527	GPL355	8	25			

### 3.2 Differential gene expression between normal and cervical cancer

**FIGURE 1:**
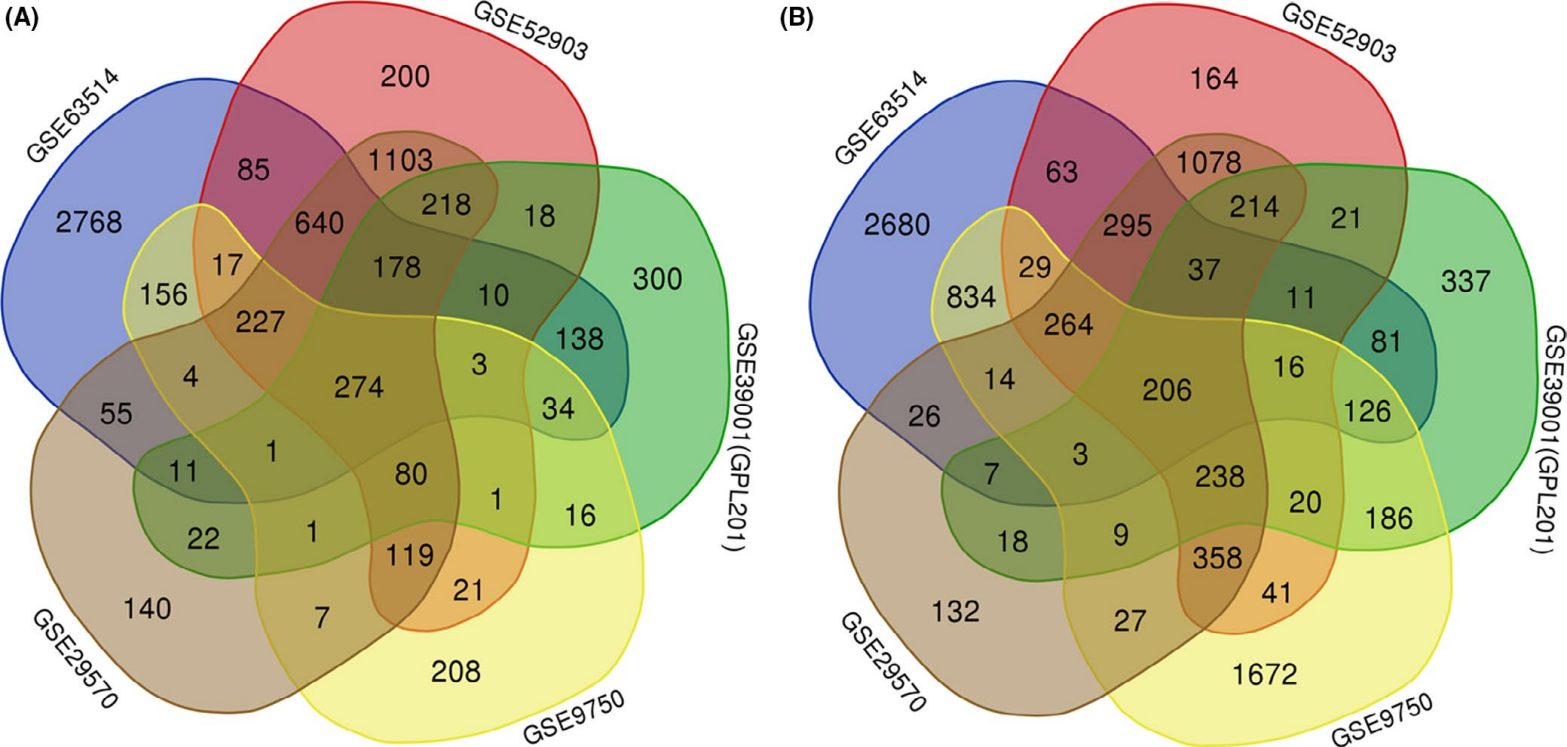
Differential gene expression during carcinogenesis in the five GEO datasets. (A) The total up‐regulated genes. (B) The total down‐regulated genes

**FIGURE 2:**
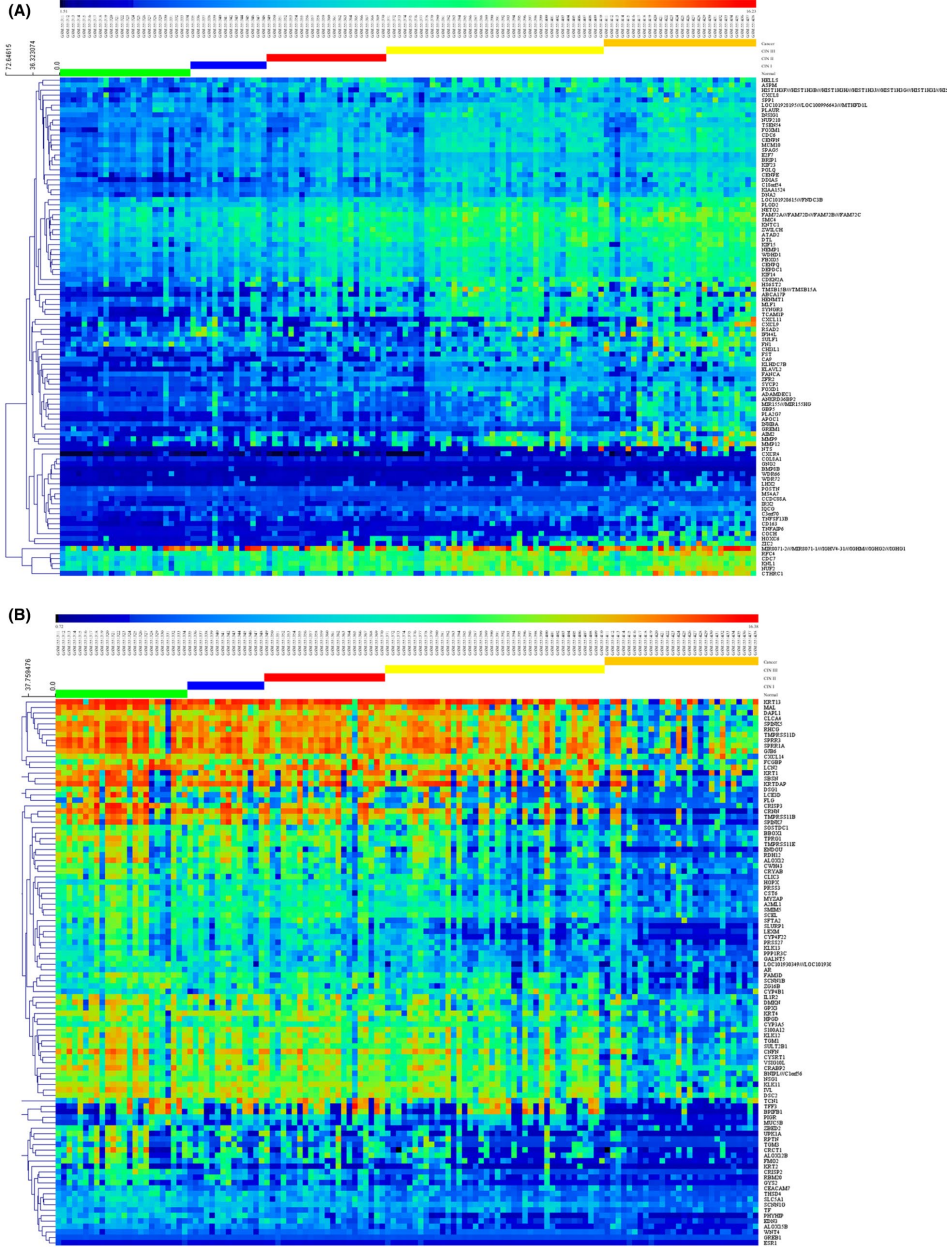
Heatmap of differential gene expression of GSE63514 in the normal cervix, CIN1, −2 and −3 lesions, and cancer. (A) The top 100 up‐regulated differentially expressed genes from 276 genes in the Intersection of five GEO datasets. (B) The top 100 down‐regulated differentially expressed genes from 206 genes in the Intersection of five GEO datasets

For comparisons among normal and cancer tissues, we analyzed all genes of normal and cancer samples online with GEO2R and tested the differences between the two categories in the five datasets we selected, after that we downloaded corresponding result files that differentially expressed genes, and gathered intersections by an online tool (http://bioinformatics.psb.ugent.be/webtools/Venn/) to make venn diagrams of genes with significantly (adjustment *p* value <0.05) high and low expression in the five datasets (Figure 1). The intersection contains 274 high expression genes and 206 low expression genes in five datasets. For the specificity of GSE63514, we further analyzed the intersections in GSE63514, and gathered top 100 gene of high and low expression in GSE63514 by online tool MEV 4.9.0 (www.tm4.org) (Figure 2), and found that there are obvious tendency in up‐regulation and down‐regulation during cervical cancer carcinogenesis. These results indicated that the predominantly differential genes in different histological grades may be important during cervical cancer carcinogenesis.

### 3.3 Differential gene expression in different histological grade of cervical cancer

**FIGURE 3:**
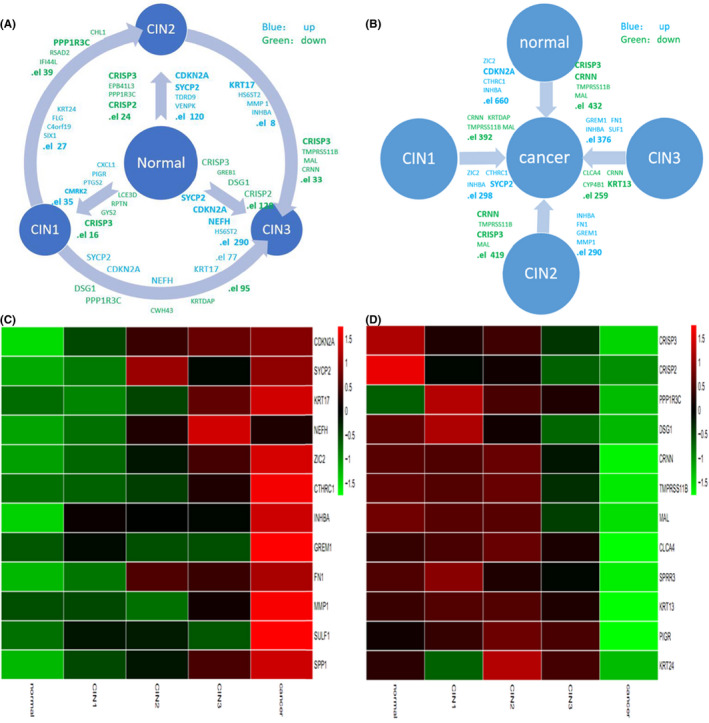
Differential gene expression during carcinogenesis. (A) The total and top 4 significantly differentially expressed genes from inter‐comparisons between the normal cervix and CIN1, −2 and −3 lesions. (B) The total and top 4 significantly differentially expressed genes in cervical cancer compared to normal tissue and CIN1, −2 and −3 lesions. Blue represents up‐regulation; green represents down‐regulation. (C) Heatmap presentations of gene expression levels of 12 genes with predominantly increasing expression level. (D) Heatmap presentations of 12 genes with predominantly decreasing expression level. Red color represents the highest expression; green color represents the lowest expression

To further find the useful biomarker, we used R statistical programming and BiocLite of Bioconductor to examine the data in GSE63514 and found the top genes to be differentially expressed in various histological grades of cervical cancer. According to the amplitude of variation with grade, all the genes with significant changes are shown in Figure 3A,B. Affymetrix Human Genome U133 Plus 2.0 Array has complete coverage of the Human Genome U133 Set, with over 47,000 transcripts. Comparing CIN1 with normal tissue, there were 35 genes that were significantly up‐regulated and 16 genes that were significantly down‐regulated. In the comparison of CIN2 with normal tissue, 120 genes were significantly up‐regulated and 24 genes were significantly down‐regulated. The CIN3 comparison with normal tissue revealed 290 genes that were significantly up‐regulated and 128 genes that were significantly down‐regulated. When comparing CIN2 with CIN1, we found 27 genes that were significantly up‐regulated and 39 that were significantly down‐regulated. In the CIN3 versus CIN1 comparison, 77 genes were significantly up‐regulated and 95 genes were significantly down‐regulated, and in the CIN3 versus CIN2 comparison, 8 genes were significantly up‐regulated and 33 genes were significantly down‐regulated. Compared with normal tissues, tumor tissues showed 660 significantly up‐regulated genes and 432 significantly down‐regulated genes. Compared with CIN1, 298 genes were up‐regulated and 392 genes significantly down‐regulated in tumors. Compared with CIN2, 290 genes were significantly up‐regulated and 419 genes down‐regulated in tumors. Compared with CIN3, 376 genes were significantly up‐regulated and 259 genes down‐regulated in tumors. These results indicate that the predominantly differentially expressed genes in different histological grades may be important during cervical cancer carcinogenesis (Figure 3A,B).

To screen the differential expression of genes at different histological grade, we further used R‐heatmap for cluster analysis and visualized the top 12 up‐regulated genes (CDKN2A, SYCP2, KRT17, NEFH, ZIC2, CTHRC1, INHBA, GREM1, FN1, MMP1, SULF1, SPP1) (Figure 3C) and down‐regulated genes (CRISP3, CRISP2, PPP1R3C, DSG1, CRNN, TMRSS11B, MAL, CLCA4, SRPP3, KRT13, PIGR, KRT24) during carcinogenesis (Figure 3D).

### 3.4 Immunohistochemical staining profile of the top genes

To confirm the significance of the up‐regulated and down‐regulated genes, we used the IHC staining to determine the 4 top up‐regulated genes (CDKN2A, KRT17, SYCP2, NEFH) and the 4‐top down‐regulated genes (CRISP3, CRISP2, DSG1, PTG) for verification of protein expression, and Ki67 as control. All eight genes, the positive responses were not good as shown in Table 3, only the repeatability of KRT17 and CRISP2 were good. These results suggested that KRT17 and CRISP2 may be used to differential different histological grades.

**TABLE 3:** The frequency of immunohistochemical staining reactivity of top gene in cervical intraepithelial neoplasm and cervical carcinoma

	n	Ki67 (%)	CDKN2A (%)	KRT17(%)	SYCP2 (%)	NEFH(%)	CRISP3(%)	CRISP2(%)	DSG1(%)	PTG (%)
Normal	15	54.5	0	0	42	40	50	100	25	0
LSIL	19	64.7	25	100	75	67	0	100	60	33
HSIL	18	100	25	90	50	38	0	95	40	0
SCC	17	100	50	92	86	60	0	93	57	0

### 3.5 Gradual Up‐regulation of Ki67 with tumor progression

**FIGURE 4:**
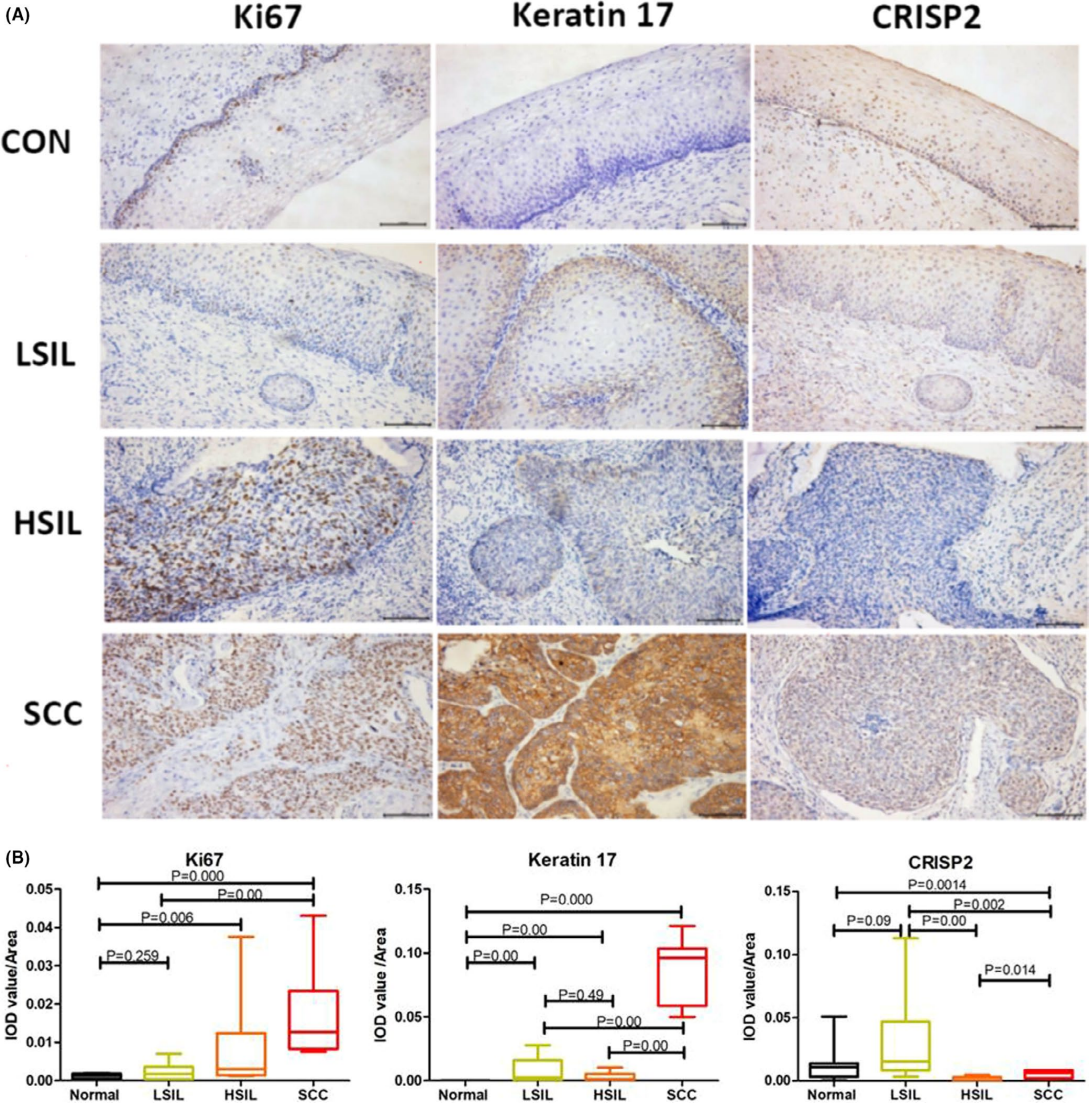
Representative immunohistochemical (IHC) staining (A) and semiquantitative analysis (B) of Ki67, KRT17 and CRISP2 during carcinogenesis. Bar scale: 50 μm. IHC results are presented with IOD value/area in different groups. The Kruskal–Wallis rank test was performed on the IHC results and *p* < 0.05 was regarded as statistically significant

Ki‐67 is a nuclear protein that is expressed in G1, S, G2 and M phases of the cell cycle and is then rapidly catabolized at the end of M phase and not detectable in cells in G0 and early G1. Ki‐67 is commonly used as a marker of cell proliferation. P16(+)/Ki67(+) nuclei have been classified with respective precisions of 77.1% and 82.6%.^10^ As the specificity of p16/Ki67 dual staining for HSIL/CC is 90.9% and 72.1%,^11^ Ki67 expression is also associated with the lesion grade of CIN.^12^ Many studies have indicated that cell proliferation in cancer patients and activation of Ki67 leads to cervical cancer progression.^13^ Therefore, Ki67 can be used as a positive control for auxiliary pathological staging diagnosis, and we evaluated Ki67 protein expression in normal, LISL, HISL and SCC samples by IHC. As shown in Figure 4, Ki67 was expressed in the nucleus, the intensity increased with histological cancer progression and the quality increased following the histological progress. Significantly high expression of Ki67 in cervical SCC was observed, and these results were consistent with those of previous reports.^13^, ^14^, ^15^, ^16^

### 3.6 Specific high expression of KRT17 in cervical cancer and non‐expression in normal tissue

KRT17, a stem cell marker, is also a positive marker used for grading CIN. KRT17 immunostaining differs according to the degree of cervical intraepithelial lesions and SCC, and surprisingly, staining is significantly correlated with increasing lesion grade of CIN and SCC.^17^ Additionally, KRT17 and p16 expression patterns have been used to distinguish (atypical) immature squamous metaplasia from high‐grade cervical intraepithelial neoplasia (CIN III).^18^ As shown in Figure 2B, KRT17 was mainly distributed in the cytoplasm. Normal squamous epithelium, normal cervical gland epithelium and mature squamous metaplastic cells were negative for KRT17 staining. KRT17 was also associated with increasing lesion grade in cervical intraepithelial lesions, as follows: LSIL>HSIL>SCC. KRT17 was extremely over‐expressed in SCC samples but was barely expressed in normal samples, which corresponded to the results of gene expression analysis. Expression of KRT17 was not significantly different in LSIL and HSIL (Figure 4). Therefore, KRT17 may be a good biomarker for cervical cancer and a lack of KRT17 expression strongly suggests a normal cervix.

### 3.7 Specific low expression of CRISP2 in HSIL

CRISP2, previously known as testis‐specific protein 1, belongs to a family of cysteine‐rich secretory proteins (CRISPs) and is strongly expressed in male reproductive tissues, including the testis, prostate and epididymis.^19^ CRISP2 mediates spermatogenesis, modulation of flagellar motility, acrosome reaction and gamete fusion.^20^ CRISP2 is mainly expressed in the cytoplasm of the lesion but is localized around the nucleus. As indicated in Figure 4, CRISP2 was significantly highly expressed in LSIL but less expressed in HSIL, which corresponds to the results of gene expression analysis. In contrast to its expression in other histological grades, CRISP2 is a specific lowly expressed biomarker for HSIL.

### 3.8 Repeatability of the expression of KRT17 and CRISP2 in other GEO datasets

**FIGURE 5:**
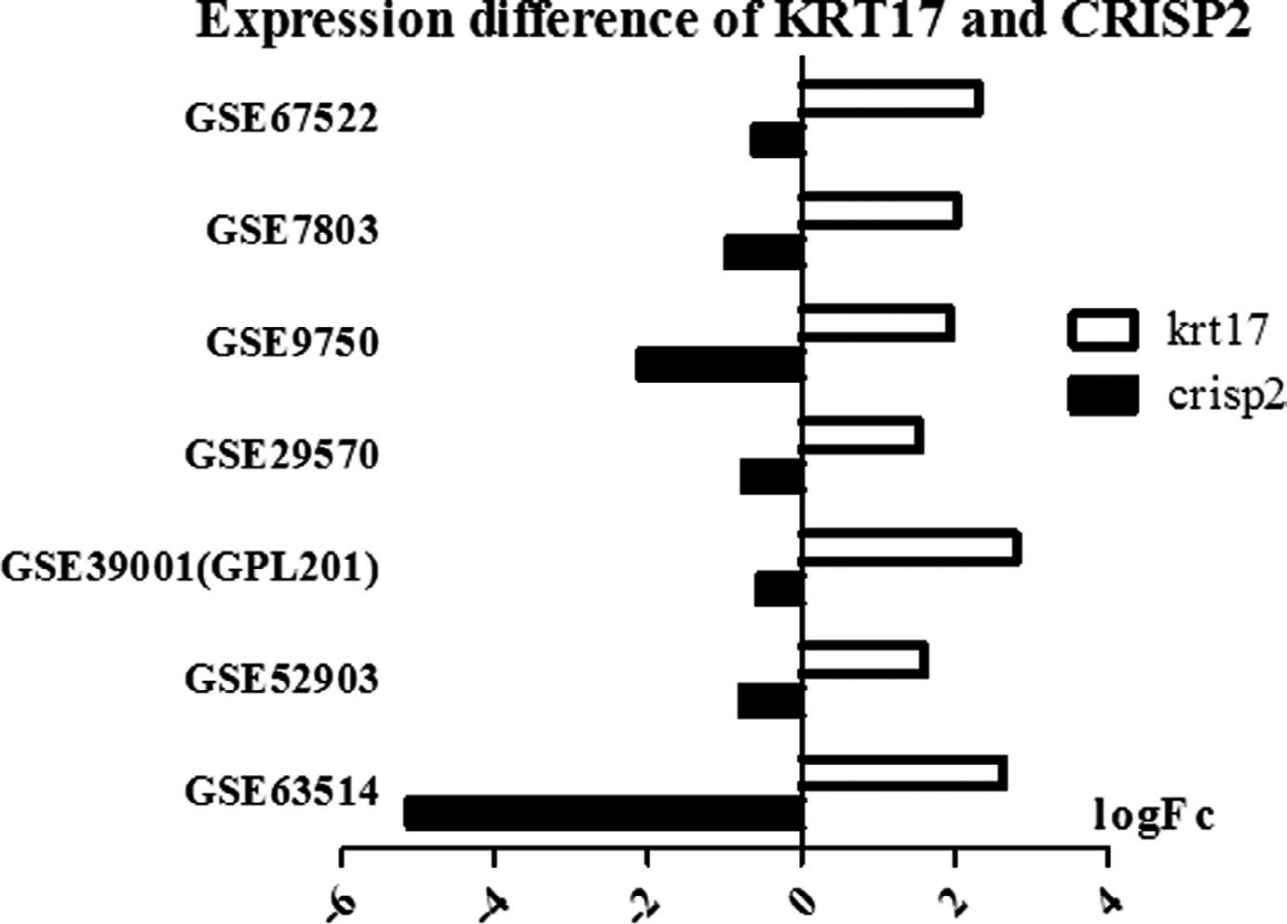
The expression of KRT17 and CRISP2 in the GEO datasets of cervical cancer. Log‐Fold changes of biomarker candidates are shown. Left is down‐regulation, and right is up‐regulation

To confirm the significance of KRT17 and CRISP2 during the period of tumor progression, we further analyzed their expression among normal and cancer samples in 6 datasets with larger sample size. The extracted data of these two genes’ expression in GEO datasets suggested the same tendency (Figure 5), so that means that combination of KRT17 and CRISP2 may be used as diagnostic biomarker to distinguish the different histologic grades of cervical cancer.

## 4 DISCUSSION

In this study, we analyzed GEO microarrays to identify differentially expressed genes as candidate targets for further IHC confirmation. We found that the expression of Ki67 was increased according to histopathological stage, whereby high expression of KRT17 was specifically found in cervical cancer and low expression of CRISP2 was specifically observed in HSIL. Therefore, we proposed that combined analysis of KRT17 and CRISP2 expression to determine different histological stages of cervical cancer, it would be helpful for accurate histological diagnosis.

Ki67 is a well‐known proliferation marker used for the independent evaluation of the prediction of cancer progression.^21^ Oncoprotein E7 of HPV inactivates the function of pRb, leading to P16ink4a accumulation.^22^ P16ink4a/Ki67 immunocytochemistry can achieve a sensitivity of 86% for LSIL and 88% for HSIL.^23^ Accordingly, we used Ki67 as a positive control for histopathological staging in our study and compared it with our novel biomarkers, but the obvious insufficiency of Ki67 is that it cannot recognize the different histological stages of cervical cancer. Therefore, more effective and affordable screening methods are needed. Protein‐based biomarkers, such as SCC‐Ag,^6^ p16,^24^ CEA,^25^ CYFRA,^26^ sCD44, MMP‐9,^27^ GINS2,^28^ HBXIP ^29^ and Pin1 ^30^ had reported, are relevant to cervical cancer. Unfortunately, most of them are applied for prognosis or efficacy evaluation, whereas specific biomarkers for various histological stages of cervical cancer for early diagnosis and treatment remain unknown.

During the progression of cervical cancer, from normal cervical tissues to CIN 1, 2 and 3, the gradually down‐regulated genes we confirmed were CRISP3, CRISP2, PPP1R3C, and DSG1. CRISP2 (gene ID: 7180) also belonging to the CRISP family, is strongly expressed in male reproductive tissues, including the testis, prostate and epididymis,^31^ mediating the adhesion between cells and cells, and participate in the fertilization process together with CRISP1,^32^ and spermatogenesis, modulation of flagellar motility, acrosome reaction and gamete fusion.^33^ Those results suggested that the decrease in CRISP2 expression may lead to decreased sperm motility. As there is no evidence of a connection between CRISP2 and cervical cancer to date, so, our finding that CRISP2 is specifically expressed at low levels in HSIL is novel.

During the progression of cervical cancer, from normal cervical tissues to CIN 1, 2 and 3, the up‐regulated genes we confirmed were SYCP2, NEFH, CDKN2A and KRT17. KRT17 (Gene ID: 3872) is located on chromosome 17q21.2, encoding keratin, type I cytoskeleton 17 (Keratin, type I cytoskeletal 17, UniProtKB‐Q04695), expressed in the outer root sheath and medulla region of hair follicles, where it is involved in the formation and maintenance of various skin appendages.^19^ KRT17 is considered a marker of basal cell differentiation in complex epithelia.^34^ The absence of KRT17 delays basaloid follicular hamartoma tumor initiation and growth in mice with constitutive Hh signaling in the epidermis.^20^ KRT17 is also associated with cell polarization and the recruitment of effector immune cells to lesion‐prone cervical epithelia. Escobar‐Hoyos et al. found KRT17 to be highly expressed in premalignant and malignant squamous lesions of the cervix,^7^ and Kim et al. showed KRT17 to be differentially expressed in adenocarcinoma and SCC.^35^ Our results are consistent with the results of those studies. Most importantly, we found that expression of KRT17 was stronger than that of Ki67 in cervical cancer, indicating that KRT17 is a more specific and suitable marker for cervical cancer.

During the progression of cervical cancer, other up‐regulated genes, such as CRISP3 (gene ID: 10321) belongs to the family of cysteine‐rich secreted proteins (CRSP), which is involved in the defense response against foreign material damage and the natural immune response of the reproductive system. CRISP3 has been found to be a prominent marker of prostate cancer gene expression,^36^ ovarian cancer.^37^ Ko WC et al. found that CRISP3 copy number was down‐regulated in oral squamous cell carcinoma T1/T2 phase, and did not change in T3/T4 phase.^38^ PPP1R3C (PTG, Gene ID: 5507) is highly methylated in colon cancer and is a feature of colon cancer epigenetics, which may play an important role in tumor cell growth associated with blood glucose levels.^39^ Desmoglein‐1 (DSG1) (Gene ID: 1828) is associated with severe dermatitis, multiple allergies and metabolic wasting syndrome.^40^ Negative expression of DSG1 on the cell membrane can be used as a marker for anal squamous cell carcinoma.^41^

During the progression of cervical cancer, other down‐regulated genes, such as synaptonemal complex protein 2 (SYCP2)(Gene ID: 10388) has been found that it is expressed uncontrolled in early oral cancer of human HPV infection,^42^ and SYCP2 gene alternative splicing events may be involved in the occurrence and development of uterine CSCC, and can be used as a bio‐diagnostic marker for CSCC.^43^ NEFH (Gene ID: 4744) involved in the generation, development and regeneration of axons. NEFH highly methylated esophageal squamous carcinoma cells can be destroyed by specific inhibitors of downstream signaling pathways.^44^ Cycle‐dependent kinase inhibitor 2A (CDKN2A) (Gene ID: 1029) controls G1 phase by regulating CDK4 and P53, is an important tumor suppressor gene. Recently, Viloria ME et al. reported that the decreased frequency of p16 (CDKN2A) expression accompanied by decreased frequency of TGF‐β1 in CIN III and cancer.^45^ This report suggested that CDKN2A could be involved in the neoplasia progression, as we predicted before. In our study, we found that all of them regulated in cervical cancer. But their reactivity in our study is no good. There exists internal and external reason. For those analysis are based on gene chips, no protein analysis, the internal reason may threaten the validity of this study, such as post‐transcriptional modification of protein, and system error of the determination of gene chips. The external reason maybe change the validity of this study, such as our experimental conditions, antibody batches, etc. Although we did not confirm their expressions, its do not hinder them to be the candidate biomarker in the future.

In summary, using a gene‐level to protein‐level analysis, our results demonstrate that KRT17 and CRISP2 are specifically expressed in various histological stages of cervical carcinoma. Therefore, combined analysis of KRT17 and CRISP2 expression is greatly helpful for early histological diagnosis of cervical precancerous lesions. Some novel methods may provide more convenient indicators, including serum biomarkers, circulating auto‐antibodies against P16,^46^ and Th2 and Th3 cytokines.^47^

## DISCLOSURE

The authors declare no conflict of interest.

## AUTHORS’ CONTRIBUTIONS

LZG participated in all the experiments, interpreted the result and was responsible for the approval of the Ethics Committee of the Women's Hospital of School of Medicine. CJH contributed to the collection and diagnosis of clinical cervical tissue specimens and interpreted the results. ZSB contributed to sectional GEO datasets analysis and write sectional manuscript. LYJ contributed expertise regarding immunohistochemical staining. ZJ participated in image production. LJH and THF conceived of all the experiments and participated in the interpretation of the results and manuscript production.

## ETHICS APPROVAL AND CONSENT TO PARTICIPATE

The collection of human samples from patients obtained the consent from the Medical Ethics Committee of Woman's Hospital, and all patients signed the informed consent for research.

## CONSENT FOR PUBLICATION

All patients signed the informed consent for publication.

## Data Availability

The datasets used and analyzed during the current study are available from the corresponding author upon reasonable request.
